# Targeting the Human Gut Microbiota—Between Conventional Therapy and Precision Genetic Engineering

**DOI:** 10.3390/nu18121958

**Published:** 2026-06-17

**Authors:** Naomi-Adina Ciurea, Laura Mahdi, Annarita Graziani, Agostino Di Ciaula, Piero Portincasa, Mohamad Khalil

**Affiliations:** 1 Department of Internal Medicine, “George Emil Palade” University of Medicine, Pharmacy, Science and Technology of Targu Mures, 540139 Targu Mures, Romania; naomi-adina.macaru@umfst.ro; 2 Doctoral School of Medicine and Pharmacy, “George Emil Palade” University of Medicine, Pharmacy, Sciences and Technology of Targu Mures, 38 Gheorghe Marinescu Street, 540139 Targu Mures, Romania; 3 Clinica Medica A. Murri, Department of Precision and Regenerative Medicine and Ionian Area (DiMePre-J), University of Bari Aldo Moro, Piazzale G. Cesare 11, 70124 Bari, Italy; laura.mahdi@uniba.it (L.M.); agodiciaula@gmail.com (A.D.C.); mohamad.khalil@uniba.it (M.K.); 4 Institut AllergoSan Pharmazeutische Produkte Forschungs- und Vertriebs GmbH, 8055 Graz, Austria; graziani@allergosan.at

**Keywords:** gut microbiota, dysbiosis, microbiome therapeutics, short-chain fatty acids, fecal microbiota transplantation, engineered probiotics, synthetic biology, MASLD, inflammatory bowel disease

## Abstract

The gut microbiota is increasingly examined as a therapeutic target because it contributes to epithelial barrier integrity, microbial metabolite production, bile acid transformation, immune regulation, and communication between the gut and distant organs. This structured narrative review synthesizes evidence on microbiota involvement in metabolic, gastrointestinal, hepatic, cancer, and neuroimmune conditions, including MASLD/MASH, inflammatory bowel disease, irritable bowel syndrome, obesity, type 2 diabetes, hypertension, colorectal cancer, Parkinson’s disease, and autism spectrum disorder. Across these conditions, microbiome findings are biologically plausible but heterogeneous. Many associations are shaped by diet, geography, medication exposure, host genetics, disease stage, sampling methods, and analytical pipelines. Microbial alterations should therefore be interpreted as context-dependent signals and candidate modifiers rather than universal causal markers. Conventional microbiota targeted strategies include diet, physical activity, prebiotics, probiotics, synbiotics, postbiotics, and fecal microbiota transplantation. These approaches are clinically familiar, but their effects are often broad, host specific, strain dependent, and difficult to assign to one mechanism. Fecal microbiota transplantation has the clearest clinical role in recurrent *Clostridioides difficile* infection, while evidence for most other indications remains inconsistent. Engineered microbial therapeutics offer greater experimental precision through signal sensing, payload delivery, metabolic modulation, and genetic circuit design. However, most evidence remains preclinical or early translational. Progress requires stronger human trials, standardized methods, mechanistic validation, safety monitoring, ecological containment, transparent reporting, and proportionate regulation.

## 1. Introduction

The gastrointestinal tract is not a static tube through which food passes but is rather a dynamic host–environment interface that is crucial to the processes of food intake and digestion [1]. Physiologically, it is from the oral cavity to the anus and covers the pharynx, esophagus, stomach, small and large intestine, rectum, and anal canal [1]. The liver, gall bladder and pancreas are accessory organs of digestion, rather than a part of the gastrointestinal tract, and are therefore treated as accessory digestive organs, which are important in regulating the flow of food through the tube, the secretion of bile, enzymes, and metabolic homeostasis rather than in the process of digestion itself [2]. This separation, however, is not just anatomical. The microbiome research, however, shows that every part of the gut has a unique pH, oxygen, transit time, exposure to substrates, immune surveillance and microbial density, which creates unique ways to interpret host–microbe interactions [3,4].

The gut microbiota is the community of living microbes present in the gut environment and the microbiome is more the collection of microbes, their genomes, metabolites, structure and interactions in a given habitat [5]. Although bacteria dominate much of the current literature, the intestinal ecosystem is not exclusively bacterial. Fungi, protozoa, and helminths are eukaryotic, whereas bacteria and archaea are prokaryotes [6]. Bacteriophages and other viruses should be mentioned separately, as they affect microbial ecology through the predation of bacteria, restructuring of communities and horizontal gene transfer of genes, but not as cellular microbes [7,8]. Accurate terminology, therefore, is crucial, as it can result in mechanists overstating, particularly with reference to dysbiosis, activation of the immune system, and microbiota-targeted therapies.

However, the recent literature cautions for more careful use of descriptions of the microbiota with numbers. Older descriptions often repeated that the human body contains “over 100 trillion” microorganisms, but revised estimates place the bacterial burden much closer to the number of human cells than earlier 10:1 claims suggested [9]. The point is not that the microbiota is insignificant, but that it is not possible to downplay its importance while relying on easy-to-remember estimates that are not precise. Instead, the importance of the microbiota for clinical purposes is not related to the amount of microbes, but rather to the metabolic capacity, ecological resilience, interindividual variability and interaction with host physiology [10,11].

The gut microbiota is known to play a role in the host’s metabolism, integrity of the epithelial barrier, regulation of the mucosal immune system, transformation of bile acids, fermentation of dietary substrates, and production of bioactive metabolites, including short-chain fatty acids [12,13,14]. These functions put the intestine and extraintestinal organs at gut–organ axes, such as the gut–liver, gut–brain, gut–immune, and gut–muscle axes [10,11]. The concept of a “healthy” microbiota is context-specific, however. A resilient microbiota does not have a single microbial fingerprint, but rather a functional stability and metabolic adaptability, colonization resistance and appropriate immune tolerance of the host [10,11]. Eubiosis, therefore, is not a clearly defined taxonomic formula but a state of functioning of the ecology [10].

Dysbiosis is similarly complex. This can include decreased microbial diversity, increased pathobionts, loss of beneficial metabolic functions, changes in bile acid metabolism [15], impaired short-chain fatty acid production [16], decreased epithelial barrier integrity or immune signaling disruption [10,11,12]. However, dysbiosis does not necessarily mean that it is the direct cause of disease. Factors such as diet, geographical location, exposure to medications, age, host genetics, disease severity, sampling method, sequencing platform, and statistics are all factors that can impact human microbiome studies [17,18]. Thus, microbial differences in metabolic dysfunction-associated steatotic liver disease (MASLD), previously described as non-alcoholic fatty liver disease (NAFLD), inflammatory bowel disease, irritable bowel syndrome, cardiometabolic disease, colorectal cancer and neuroimmune disorders could be causal contributors, secondary effects, compensatory responses, or disease-context markers [10,18,19]. The 2023 multisociety Delphi consensus supports the use of MASLD and metabolic dysfunction-associated steatohepatitis (MASH) as the preferred terms, while NAFLD and NASH are only used when referring to older literature and historical terms [20].

Hence, this review explores the gut microbiota as a therapeutic target in both conventional and precision therapeutic strategies. Common treatments are diet, exercise, prebiotics, probiotics, synbiotics, postbiotics and fecal microbiota transplantation (FMT). Three types of precision approaches are engineered probiotics, synthetic biology-based microbial circuits and disease-responsive live biotherapeutics [21]. The review emphasizes the distinction between human evidence and animal and in vitro data, associations and causal relationships, established clinical interventions and emerging or speculative interventions throughout, as recently requested for better causal reasoning in microbiome research [18].

## 2. Literature Search Strategy and Evidence Evaluation

This article was developed as a structured narrative review rather than a systematic review or meta-analysis. The goal was not to generate a “pooled” quantitative estimate, but to integrate existing knowledge and evidence on the gut microbiota as a therapeutic target from conventional microbiota-modulating strategies and emerging engineered microbial strategies. A structured narrative approach was deemed appropriate for the field, as it involves different study designs, disease contexts, sequencing methods and intervention models and allows for integration of clinical, translational and mechanistic evidence within a framework that applies explicit rules for source selection and interpretation.

Literature search was performed in PubMed/MEDLINE, Scopus, Web of Science, and Google Scholar for additional checking. Literature searches focused on the last 10 years of published literature, with greater weight on recent studies, systematic reviews, meta-analyses, consensus statements, randomized trials, and high-quality translation studies. Only older landmark studies were retained if they contained historically necessary definitions, fundamental mechanisms or generally accepted conceptual frameworks that were not available in more modern studies.

A search strategy was developed that used words associated with microbial ecology, disease mechanisms and therapeutic interventions. Some of the most important search terms were: “gut microbiota”, “gut microbiome”, “dysbiosis”, “intestinal permeability”, “gut barrier”, “short-chain fatty acids”, “bile acids”, “lipopolysaccharide”, “metabolic endotoxemia”, “MASLD”, “MASH”, “NAFLD”, “NASH”, “inflammatory bowel disease”, “IBD”, “irritable bowel syndrome”, “IBS”, “celiac disease”, “*Clostridioides difficile infection*”, “colorectal cancer”, “probiotics”, “prebiotics”, “synbiotics”, “postbiotics”, “fecal microbiota transplantation”, “engineered probiotics”, “live biotherapeutic products”, “synthetic biology”, and “genetically engineered microorganisms”. When using terms for consistency with the rest of the review, MASLD and MASH were used as preferred terms, and NAFLD and NASH were only used when referring to older literature or historical nomenclature.

The sources included were peer-reviewed human studies, randomized controlled trials, cohort studies, well-designed observational studies, systematic reviews, meta-analyses, consensus papers, mechanistic animal studies, and relevant in vitro studies. Disease associations and therapeutic relevance were discussed primarily from the human clinical point of view. Studies of animals and in vitro models had the main purpose of explaining potential mechanisms, revealing new therapeutics or elucidating pathways not directly gleaned from human studies.

The sources were excluded if they were not related to the biomedical claim being made; were not directly relevant to the cited statement; duplicated another source unnecessarily; lacked peer review; or provided only marginal relevance regarding gut microbiota, gastrointestinal disease, metabolic disease, and/or microbiota-targeted intervention. Special effort was made to not include citations that did not match the authors’ claims in the manuscript, such as those not relevant to biomedical microbiota research.

Cautious language is used to interpret the evidence, depending on the type of evidence and its strength. Human observation results are reported as associations, unless there is evidence of intervention or mechanistic evidence. Animal studies are presented as mechanistic or preclinical evidence rather than proof of human causality. For clarification of biological plausibility, in vitro studies, but not clinical studies, are used to clarify clinical efficacy. Phraseology and the use of phrases like “human observational studies suggest,” “animal models indicate,” “mechanistic studies support,” “evidence remains associative,” “causality has not been established,” and “findings vary across cohorts” is applied intentionally throughout the review to differentiate established findings from emerging or speculative interpretations.

## 3. Gut Microbiota in Health, Dysbiosis, and Host–Microbe Signaling

### 3.1. Microbiota, Microbiome, and Eubiosis

The gut microbiota is defined as the set of living microorganisms that reside in the intestinal tract, whereas the microbiome is composed of microorganisms, their genes, metabolites, structural elements and interactions with the host in the gut environment [22]. This is important as it may be possible to see a change in some bacterial genus without a significant change in the numbers. Sequencing reveals that strains can vary in the function of their genes and taxonomy, but have similar overall profiles [17].

Eubiosis is better understood as a resilient functional state than as a fixed normal composition. A healthy individual can have a different microbial profile due to the different diet, location, age, medications, immune response, and genetics of the host and the methods used to sequence the microbes [10]. The more salient question is whether the ecosystem is capable of supporting colonization resistance, epithelial integrity, nutrient metabolism, immune tolerance and host signaling. Different responses to inflammation, site, diet and disease stage are possible for the same taxon [23]. Table 1 below gives an approximate guide to the number of microbes present at various anatomical sites and the representative groups of microbes present. These values should be considered as ecological ranges and not as norms as the density and composition of microbial flora are influenced by the sampling method, oxygen tension, pH, diet, age, medications, transit time and disease state.

### 3.2. Dysbiosis as a Functional Disturbance

Although microbial imbalance is a common term used to describe dysbiosis, this is not meaningful if it simply relates to an increase or decrease in selected taxa [27]. Dysbiosis is considered more in terms of a disturbance to the composition, diversity, metabolic output, spatial organization, and host interaction [27]. It can include decreased fermentation of fiber by bacteria, increased growth of pathobionts, decreased colonization resistance, altered bile acid transformation, movement of microbes to closer proximity to the epithelium, or exaggerated immune recognition of microbial products [28]. Dysbiosis should thus be considered as an ecological and functional imbalance and not just a list of taxa [29].

This distinction is important because a lot of the disease-related microbiome findings are based on observational studies. The cross-sectional studies have the ability to reveal differences between patients and controls but are not necessarily able to determine if the differences precede the disease, follow inflammation, are due to a different medication, or reflect changes in physiology [18]. Factors that can affect reported microbial signatures include antibiotics, proton pump inhibitors, metformin, bowel preparation, stool consistency, sampling site, sequencing platform and statistical pipelines [17]. Recent consensus guidance cautions that repeated association is not enough for causality. It requires longitudinal evidence, controlled perturbation, mechanistic coherence, and careful use of preclinical models [18].

### 3.3. Mechanistic Pathways Linking Microbiota to Host Physiology

The gut barrier is one of the important barriers between the activity of the microbiota and host disease [12,30]. These consist of a mucus layer, antimicrobial peptides, epithelial cells, tight junction proteins, immune cells, vascular structures, and microbial metabolites [31]. The tight junction proteins control paracellular permeability, and mucus forms a barrier for direct contact between microbes in the lumen and epithelial cells [28]. Metabolic endotoxemia, immune activation and low-grade inflammation are linked to the presence of lipopolysaccharide in portal or systemic circulation in the event of these systems’ weakness [32]. The dysfunction of the barrier should not be considered as a one-off occurrence. Epithelial damage can lead to changes in the microbiota and to inflammation [31].

However, another pathway of communication among hosts is via microbial metabolites [33]. Short-chain fatty acids (SCFAs) are formed by the microbial fermentation of dietary fiber and host-derived substrates, primarily acetate, propionate and butyrate [34]. They impact the energy metabolism of epithelial cells, mucin production, regulation of tight junctions, differentiation of regulatory T cells, macrophage activity and inflammatory signaling [13]. They do not always have a protective effect. They have different effects depending on the context of the illness, the metabolic state of the host, the location of their action in the intestine, and concentration [35]. There needs to be a mechanism in addition to decreased short-chain fatty acid-producing taxa for the onset of disease [35].

Another host–microbe interface that is recognized as key is bile acid metabolism [36]. The liver generates primary bile acids, which are converted to secondary bile acids in the intestine by intestinal bacteria [36,37]. These molecules act via the farnesoid X receptor (FXR) and Takeda G protein-coupled receptor 5 (TGR5), which affect lipid and glucose metabolism, antimicrobial function, epithelial barrier integrity and immune responses [38]. This pathway provides a pathway to link dysbiosis with inflammatory bowel disease, MASLD/MASH, colorectal cancer and metabolic disease, but the results vary between cohorts.

These pathways provide an understanding of the importance of gut–organ axes. The gut–liver axis involves the transfer of microbial metabolites, endotoxins, and bile acids to the liver by portal circulation and can impact steatosis, inflammation, and fibrogenesis [38]. The gut–brain axis features other interactions between the gut and the brain, such as immune mediators, enteroendocrine signaling, microbial metabolites, vagal pathways and barrier integrity that facilitate the neural communication between the gut and the brain [39]. Gut muscle axis: There is emerging evidence that links microbial metabolism, sleep disruption, mitochondrial function and physical performance, although the evidence is context dependent [40]. Microbial signals modulate tolerance, inflammatory thresholds and responses to treatment, and the gut–immune axis is therefore wider.

This framework guides the disease sections that follow. Metabolic, gastrointestinal, hepatic, cancer and neuroimmune diseases are not separated from each other and are not reviewed as individual summaries. Instead, it examines recurring mechanisms across conditions, including barrier disruption, metabolite imbalance, bile acid remodeling, metabolic endotoxemia, immune activation, and gut–organ communication. This is in response to the increasingly urgent need to use functional and translational models instead of taxonomy to explain the microbiome [10]. Each section distinguishes between human findings and animal and in vitro data, as well as association and causality [18].

## 4. Microbiota in Metabolic and Cardiometabolic Disease

There are a number of common mechanisms associated with the microbiota that are shared by metabolic and cardiometabolic diseases. These include low-grade inflammation, decreased barrier function, altered microbial metabolites, bile acid signaling and host metabolic stress. Insulin resistance and cardiovascular risk are regarded second, as many people are obese before they become insulin resistant; or are insulin resistant and at cardiovascular risk at the same time; or are insulin resistant, at cardiovascular risk, and obese.

### 4.1. Obesity

Obesity is an example of how the use of only a single taxonomic parameter is insufficient to account for complex metabolic disease. Human studies have been conducted later, but there is no evidence for a higher Firmicutes-to-Bacteroidetes ratio with obesity [41,42,43]. In an evaluation of 120 metabolically healthy adults with obesity, two patterns of evolution of the extracellular vesicles from the stool were correlated with different inflammatory profiles, and the Firmicutes and Bacteroidetes patterns were inconsistent, with no specific pattern that could be directly associated with the development of obesity [42]. A review of pediatric obesity recently came to a similar conclusion with a mix of results and inconclusive correlations among the Firmicutes-to-Bacteroidetes ratio [43].

The usable one is defendable. Microbial alterations linked to obesity may include changes in energy harvest, intestinal permeability, bile acid metabolism, production of short-chain acids, inflammation in the adipose tissue and insulin sensitivity [38,44,45]. All of these mechanisms lend a degree of biological plausibility, but cannot be considered direct proof of the initiation of obesity due to dysbiosis [18]. Both diet and geography, as well as medication use, sequencing method, host genetics and metabolic phenotype can influence the observed microbial patterns [17,18,46]. Therefore, the conclusions of human microbiome studies in obesity should be considered as contextual markers of metabolic status, inflammatory markers and cardiometabolic risk, and not as a single microbial cause of obesity [42,44]. The most compelling idea is that the microbiota can affect metabolic risk for obesity, although the causal direction and the predictability of therapeutic efficacy is still unclear [18].

### 4.2. Type 2 Diabetes

Hyperglycemia, insulin resistance, adiposity, dietary factors and exposure to medications may be linked, so a pathway interpretation of the microbiome evidence is needed for type 2 diabetes. The metabolites produced by the gut microflora can exert a negative impact on host glucose metabolism indirectly by entero-endocrine signaling, bile acid receptor signaling, production of short-chain fatty acids, changes to the intestinal barrier, and low-level inflammation [47,48]. In addition, it is possible to consider the development of type 2 diabetes as a metabolic disease that is linked to in vivo interaction between microbial metabolites, barrier disruption and inflammatory signaling, as well as the host’s glucose regulation, but not solely by an endocrine pathway [49]. The microbiota must thus be considered as an integral component of a host metabolic network but not as an autonomous entity that regulates the blood glucose [48,49].

The taxonomic results in type 2 diabetes are only useful when considered as associative and study-dependent. Escherichia Shigella was the most consistently found taxon in type 2 diabetes in a systematic review of 58 observational studies, whereas Faecalibacterium prausnitzii seemed to be more commonly found as a potentially protective butyrate-producing organism [50]. The same review found that they could not make causal inferences due to heterogeneity and the design of the observational studies [50]. This constraint is clinically relevant as it can alter the microbial pathways that are associated with diabetes, due to exposure to medications [50,51,52]. The MIGHTY randomized clinical trial showed that the use of metformin alone and in combination with liraglutide resulted in a shift towards bile acid and short-chain fatty acid (SCFA)-producing gut microbial taxa in the case of youth-onset type 2 diabetes [53]. This microbiome pattern in treated diabetes could thus be related to both the disease and the drug treatment [53].

The same should be applied to carbohydrate transport pathways that are dependent on microbes. Sequencing is able to detect the capacity of the microbial community, but not necessarily to predict that glucose uptake by microorganisms leads to hyperglycemia in the host [17]. An indirect calculation is a more reasonable one. Short-chain fatty acids, bile acids, exposure to endotoxin, changes in epithelial permeability and inflammatory signaling may be altered by microbial metabolism [48]. The pathways, in turn, can affect insulin sensitivity and glucose homeostasis [48,49]. It is now known that the microbiota is not a direct cause of type 2 diabetes, but instead is a modifiable factor of glucose homeostasis [18,50]. A microbiota-directed intervention could influence glycemic markers by repairing the barrier, reducing inflammation, changing bile acid signaling, changing diet, or drug interactions, but without being able to demonstrate any specific diabetic microbial signature [48,52].

### 4.3. Hypertension

Instead, hypertension should be considered a cardiometabolic disorder where the signals from the gut can interact with the vascular, renal, immune and neural pathways. In the American Heart Association advisory, the gut microbiome is identified as a nascent field of hypertension research that holds promise, but less so for clinical use. This caution is needed because sodium consumption, renal function, vascular stiffness and medication all affect blood pressure.

There are several possible mechanisms, none of which would be a single diagnostic sign. The mechanisms by which short-chain fatty acids could modulate blood pressure include effects on the vascular tone, renal signaling, inflammation and sympathetic pathways [54,55]. In older adults in China, there were associations of SCFAs with blood pressure in a cross-sectional study, which did not allow for determining directionality [56]. The host and microbial metabolism of dietary precursors like carnitine and choline can also lead to the generation of trimethylamine *N*-oxide (TMAO) which is correlated with cardiovascular dysfunction, but also influenced by diet and renal clearance [57].

A link between diet, microbiota and immune activation is salt sensitive hypertension. Evidence for the effects of high sodium intake on microbial ecology, availability of short-chain fatty acids (SCFAs), inflammatory signaling, sympathetic activity, and renal sodium handling are reviewed and suggest that much of the evidence is preclinical or early translational [58,59]. The unbalanced point is that dysbiosis has been linked to hypertension and has been proposed to play a role in this disease, potentially via metabolites, barrier dysfunction, immune activation, sympathetic signaling and sodium handling. It should not be presented as a direct cause.

## 5. Microbiota in Gastrointestinal and Liver Disease

### 5.1. Inflammatory Bowel Disease

The microbiome relevance of inflammatory bowel disease (IBD) is not only reflected by the definition of the disease but also in the relationship between the microbiome and the integrity of the epithelial barrier and the immune system in the mucosa [60,61]. An IBD dysbiosis has been described recently as a decrease in diversity, a loss of commensal metabolic functions, and an increase of pathobionts, but the signature varies depending on the site, activity, and treatment, as well as the sampling method [62,63]. This variation renders the taxonomic interpretation a bit shaky. A more useful question would be whether microbial change comes to a common theme of maintaining inflammation. The vast majority of the evidence for the human microbiome in IBD is observational or, like generating a synthetic microbiome from the literature, synthesis-based, and so depletion of diversity, of SCFA-producing taxa, and expansion of pathobionts should be viewed as patterns that occur during the course of the disease, not as diagnostic or causal signatures [62,63].

The robust trend is not the reoccurrence of one organism, but the convergence of the shifts in microbes and barrier and immune dysfunction. Some studies have reported decreased levels of short-chain fatty acid (SCFA)-producing bacteria, including Faecalibacterium, and increased levels of facultative anaerobes like *Escherichia coli* in IBD patients [64,65]. Clinically important patterns include supporting energy metabolism and regulatory immune function of the intestinal epithelia with butyrate, and the possibility of adhering to epithelial cells and surviving in macrophages and enhancing inflammatory signaling in Crohn’s disease with adherent invasive *E. coli* [66]. Despite this, there is no evidence that these associations mean either organism is the cause of IBD. Inflammation may itself alter oxygen availability, the composition of the mucus, and the gradients of nutrients and restrict access to oxygen, which favors facultative anaerobes and restricts strict anaerobes [67]. Microbiota and inflammation must thus be considered as a feedback loop and not a unidirectional causal loop [18,32].

The dysfunction of the barrier is the best link between dysbiosis and mucosal disease [68]. Defects in organization of the mucus, antimicrobial peptide activity and tight junction regulation in IBD can allow for increased contact between microbes and the epithelium [30,31,69]. This contact can trigger activation of pattern recognition receptors and stimulation of cytokines, such as tumor necrosis factor (TNF), interleukin 23 (IL-23) and T helper 17 (Th17) pathways [63]. This is also the case for microbial metabolites. The decreased production of butyrate, changes in the pools of bile acids and a disturbed course of tryptophan metabolism may contribute to reduced epithelial repair and immune tolerance, but the relative importance of these pathways varies between Crohn’s disease and ulcerative colitis [62,70]. These disease-specific variations do not allow for broad conclusions and are conducive to making conclusions based on mechanisms across studies, cohorts, phenotypes and over time, as well as helping to prevent premature claims of causation.

In terms of treatment, the disease of IBD shows promise and restraint. Changes in diet affect the substrates available for fermentation and immune activation and can be variable [71]. The beneficial effect of probiotics can be specific to ulcerative colitis or pouchitis [72]. FMT remains less predictable in IBD than in recurrent Clostridioides difficile infection because donor effects, engraftment, disease phenotype and safety monitoring are unresolved [63]. The concept of the use of engineered anti-inflammatory microbes and defined bacterial consortia is conceptually attractive as they would be able to provide metabolites, cytokine modulators, or barrier supportive signals more accurately, but with the majority of evidence in preclinical or early translation stages, it remains to be seen if this can be proven in humans [73].

### 5.2. Irritable Bowel Syndrome

The microbiota signal of IBS is not uniform and is a disorder of gut–brain interaction [74]. The differences in microbiota between diarrhea-predominant, constipation-predominant and mixed IBS are also different and may also vary between post-infectious IBS and IBS not triggered by an infection [75]. A narrative review suggests that the dysbiosis of IBS should be understood in terms of the barrier function, the activation of the immune system, microbial metabolites and brain signaling, in addition to taxa [32,76,77]. Post-infectious IBS supports this interpretation as the infection can lead to changes in mucosal immunity, permeability and microbial ecology, with the exception of microbiome-directed therapy which is not consistently supported in this subgroup [75].

Microbial functionality is more closely associated with symptoms like changes in bowel habit, bloating and pain than to organism lists [78]. Altered gas production from fermentation may lead to increased gas production and luminal distention, and changes in the profile of SCFAs could be a mechanism that impacts on motility, epithelial signaling, and visceral sensitivity [35,79]. Case–control and intervention data are conflicting and limited in a systematic review of fecal SCFAs in IBS, so it is unclear whether SCFAs are increased, depleted or protective in IBS patients [77,80]. The metabolism of bile acids is also subtype dependent. In IBS-D, microbial bile acid alteration may contribute to colonic secretion and motility [36,81,82] and the results are related to the stool form and handling of bile acids by the host.

The evidence of physical activity and lifestyle, motility, inflammation, and microbial ecology are interlinked, but should not be considered as disease defining. Exercise seems to have a role in altering the composition of the microbiota and symptoms but this effect is dependent on the individual and the type, intensity, duration of exercise, and IBS subtype [83]. This aligns with the broader IBS evidence base, where systematic review findings on fecal SCFAs remain conflicting and subtype dependent rather than causally settled [80]. Overall, IBS microbiome evidence can be used to support mechanism-based grouping and personalized treatment, but should not be used to make blanket statements about a causative link between dysbiosis and IBS.

### 5.3. Celiac Disease

Gluten exposure, mucosal immunity, epithelial permeability and HLA DQ2 or DQ8 susceptibility all play a part in celiac disease [84,85]. The addition of the findings regarding microbiota provides an added layer of plausibility, but not one that replaces the established gluten-induced immune pathway [86]. The authors of a scoping review of 48 microbiome studies, Luz and Pereira, determined that there was significant variation in sample type, sequencing methods, size and taxa included in the studies, thus making it challenging to find consensus on a celiac microbiome signature [87]. This renders the language used in the cause–effect sentence inappropriate.

The analytical model is a sequence model. Peptides of gluten that escape digestion in their entirety might pass through an intact epithelial barrier and get deamidated by tissue transglutaminase 2 [88]. These peptides bind to HLA DQ2 or DQ8 molecules and are more efficient at triggering gluten-reactive CD4 T cells, resulting in the production of anti-tissue transglutaminase antibodies and the formation of mucosal inflammation and villous injury [89]. Microbiota-related factors could impact this pathway through altering peptide processing, barrier and mucus integrity, the availability of short-chain fatty acids, and modulation of immune tone [90,91]. Human work indicates that the ecology of the duodenum could be different than the stool profile in celiac disease, as the duodenum is more likely to be injured than the stool profile [92,93,94].

Therefore, microbial transglutaminase can be considered as a new hypothesis [95]. It can either alter proteins or participate in the generation of neoepitopes but is not yet proven to be a disease mechanism [95,96]. Microbial enzymes have been suggested as potential influences on the exposure to antigens and immune amplification, but have not been established as direct causes of celiac disease in recent reviews [89,90].

### 5.4. Clostridioides Difficile Infection

The most straightforward clinical example of disruption of colonization resistance by antibiotics leading to an ecological niche for pathogen recurrence is in the case of *Clostridioides difficile* infection (CDI). Antibiotics decrease microbial diversity and eliminate competitors for *C. difficile* nutrition, induce bile acid modification and inhibit germination or vegetative growth of spores [97,98]. The mechanism is not simply an absence of “beneficial bacteria.” Because of the loss of community functions, the chemical ecology of the colon will change. The germinating potential of primary bile acids is suggested to stimulate *C. difficile* germination, while the germ inhibitory potential of secondary bile acids, formed by commensal bacteria, is suggested to inhibit outgrowth, depending on the strain and concentration of the bile acids and the host context [98]. This is why recurrent infections always occur after antibiotic therapy. This drug can decrease the pathogen burden, without, at the same time, possibly resolving the ecological defect.

Currently, the evidence for FMT is the strongest and follows guidelines in the treatment of recurrent CDI where the therapeutic target is a remarkably direct one: restore microbial diversity, colonization resistance and bile acid conversion following antibiotic-associated ecological disruption [99]. FMT has been recommended by the American Gastroenterological Association for specific indications in selected adults with recurrent CDI following antibiotic treatment for their disease but it is held back for other indications including IBS and IBD [99]. Another sign of this greater evidence base is FDA approval of standardized products to prevent recurrent CDI, as opposed to microbiome-related disorders more broadly [100]. Even here, FMT should not be described as risk free. The clinical implementation and regulatory review of translation is still facing major challenges like donor screening, product standardization, adverse event monitoring and immunocompromised host safety.

### 5.5. MASLD/MASH

Metabolic dysfunction-associated steatotic liver disease (MWD), also known as non-alcoholic fatty liver disease (NAFLD), and metabolic dysfunction-associated steatohepatitis (MASH), or non-alcoholic steatohepatitis (NASH), should be viewed as a gut–liver axis that does not necessarily imply a linear progression of microbial cause of disease [101]. The question is not whether dysbiosis exists. It is whether microbial functions plausibly exacerbate metabolic injury in a host which is pre-determined by insulin resistance, adiposity, diet and genetic makeup. The majority of human MASLD/MASH microbiome studies are also observational or early translational [102,103,104]. These findings may be confounded by obesity, insulin resistance, diet, medication exposure and stage of disease [103,104]. Any differences in the microbiology should thus be a mechanical signal and a candidate modifier. They are not to be used as standalone predictors for progression.

The best mechanistic connection between dysbiosis with barrier failure and hepatic inflammatory signaling comes from these two studies [105,106]. Enhanced intestinal permeability can allow lipopolysaccharide and other microbial products to enter the liver via portal circulation to activate Kupffer cells, toll-like receptor pathways, oxidative stress and fibrogenic responses [32,104,105,106,107]. The second route comes with the production of bile acid remodeling (BA), which can alter FXR and TGR5 signaling and impact lipid metabolism, glucose handling and immune regulation [38,108]. The decrease in short-chain fatty acid production might compromise the integrity of the epithelium and anti-inflammatory properties, but this is not a specific effect of MASLD and should not be overinterpreted [105].

**Figure 1:**
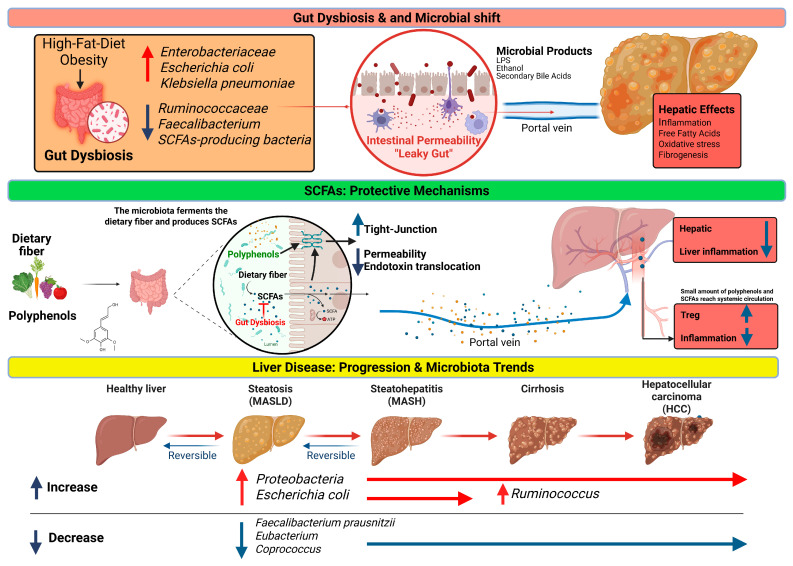
Microbiota associated gut liver mechanisms implicated in MASLD/MASH. The figure illustrates how diet, obesity, dysbiosis, intestinal permeability, microbial products, bile acid changes, SCFA-related mechanisms, and portal circulation may interact with hepatic inflammation, oxidative stress, free fatty acid accumulation, and fibrogenic signaling. The lower panel should be interpreted as a schematic representation of microbiota associated changes across MASLD/MASH severity, not as proof of a deterministic or microbiota-driven sequence from healthy liver to steatosis, MASH, cirrhosis, or hepatocellular carcinoma.

The endogenous production of ethanol, due to microbiological activity in the gut, also has to be taken into consideration, particularly in more severe steatotic phenotypes, but its clinical implication will vary depending on the context [109]. Overall, it is important to consider the alterations in microbiota as a modifier of inflammation, permeability and fibrogenesis, rather than as deterministic markers of progression from steatosis to MASH and advanced liver disease [110]. The gut–liver mechanisms discussed in this section are summarized in Figure 1, which shows a conceptual model that connects the concepts of dysbiosis, intestinal permeability, microbial products, bile acid remodeling, SCFA-related barrier support, endogenous ethanol production, inflammation and fibrogenic signaling. The figure does not imply there is a direct and inevitable link between microbiota changes and the development of MASH, cirrhosis, or HCC from steatosis.

## 6. Microbiota in Cancer and Neuroimmune Conditions

### 6.1. Colorectal Cancer

Colorectal cancer is the best example of cancer in this regard, since there is microbial evidence that could be linked to specific functions of the tumors. Enrichment of Fusobacterium nucleatum, enterotoxigenic Bacteroides fragilis and pks-positive *Escherichia coli* in selected colorectal cancers has been supported by human tumor studies, although these are not considered to be universal causes or independent markers of colorectal cancer [111]. The evidence is greatest if combined with experimental mechanisms and human tumor-associated enrichment. There is still a lack of evidence that these organisms play a role in the onset of carcinogenesis in all patients [111,112]. The best argument is mechanistic. Fusobacterium nucleatum also has a specific clade that has been found to be adapted to the colorectal cancer niche and that both suggests tumor selectivity and whether colonization is an initial cause or an exploitation of the tumor niche [112]. Bacteroides fragilis (enterotoxigenic) contributes to pathways involving epithelial barrier injury and STAT3-mediated inflammatory and proliferative signaling [113], and *Escherichia coli* with a pks gene contributes to a genotoxic pathway via its toxin colibactin’s related DNA damage. Intratumoral pks-positive E. coli has also been linked to the molecular characteristics of CRC [111].

These findings provide a biological argument for the role of microbes and do not rule it out. The location of the tumor, diet, integrity of the barrier, immune tone, and exposure to antibiotics and molecular subtype of the tumor can influence whether a microbe is a driver, amplifier or passenger [114,115,116]. This is important clinically because microbial presence does not necessarily imply risk or treatment [117]. This is the same for inflammation and barrier dysfunction. These are plausible mechanisms but may also be associated with tumor growth, exposure to therapeutic drugs or local inflammatory conditions [117]. Therefore, for strong inference, it is important to have a temporal sequence. Failing to sequence these microbes could lead to a misinterpretation of microbial correlations and causation of biology, particularly in advanced disease states [117]. This is important for microbiota-directed prevention, prognosis and adjunctive treatment approaches across diverse patient cohorts [118]. Microbes can also modulate immune checkpoint response through modulation of tumor immune tone, but this is more than just association that is required for clinical use [119]. The use of microbial markers to improve risk, prognosis or treatment stratification will only be useful if read in context of the host, tumor, treatment and microbial context [118,119].

### 6.2. Parkinson’s Disease and Autism Spectrum Disorder

Parkinson’s disease and autism spectrum disorder illustrate the need for caution with regard to gut–brain axis claims [120,121,122]. Gastrointestinal dysfunction can occur before motor symptoms due to Parkinson’s disease and there is a potential link between intestinal inflammation, alpha synuclein pathology, and vagal signaling from the gut to the brain [123,124]. Plausibility is not the issue; the direction of the effect is the issue. Microbiological changes may play a role in inflammation or in alpha synuclein processing, but can also be due to constipation, diet, medications, prodromal autonomic dysfunction, or decreased mobility [125]. Mechanistic testing is supported by animal models, which can allow for the manipulation of the microbiota to affect motor, inflammatory and metabolic phenotypes under controlled conditions [18,126,127]. These results are preliminary studies conducted in a laboratory setting. They reinforce the development of hypotheses, but they are unable to prove that dysbiosis is a cause of Parkinson’s disease in people. Long-term human studies are thus needed for translation, which include samples of prodromal stages, consider medication and constipation, and link microbial features to measurable neural or immune endpoints.

In the case of ASD, extra sensitivity is required as the phenotype is complex and the gastrointestinal tract may be affected, and a restricted diet or medication exposure may affect the microbiome’s patterns. The authors of one multi-angle meta-analysis of 690 samples found that the relative abundance of taxa might vary by age, sex and bowel function [128]. This finding weakens deterministic claims about a single autism microbiome. More recent reviews have reported on the connection between microbiota and immune, metabolic and neural pathways with behavior but also noted that the available data is mostly associative and prone to confounding [129]. Some studies suggest that microbiota-based interventions can lead to gastrointestinal and/or behavioral improvements, but there is little evidence in meta-analysis studies due to small sample sizes and inconsistent intervention and outcome measures [130]. The uncertainty does not render the field null and void. It implies that the presence of microbes should be viewed as potential indicators of the clustering of symptoms, gastrointestinal symptoms or outcomes of treatment and not as definitive causes of neurodevelopmental disorders. This is a defensible conclusion; therefore, it is very narrow. A search for microbes in Parkinson’s disease and autism could serve as a source of possible mechanistic subgroups or treatment hypotheses. Studies currently do not provide justification for the claims that dysbiosis is directly responsible for neuroimmune disease.

## 7. Conventional Microbiota-Targeted Interventions

### 7.1. Diet and Physical Activity

Dietary and activity-based interventions alter the substrates of microbial organisms and not just for a single organism. This is the one thing they lack. The effects are spread throughout the diet quality, host metabolism, microbiota, adherence and medication exposure. Dietary fiber is the most favorable starting point, as fermentation may lead to an increase in the short-chain fatty acids acetate, propionate and butyrate which have beneficial effects on energy metabolism, barrier integrity and immune regulation of epithelial cells [131]. However, there is no uniform response to fiber. The effect that a diet will have depends on its fermentability, the dose of the diet and the capacity of the resident microbes.

Mediterranean-style dietary patterns are an ecological intervention due to their combination of dietary fiber and polyphenols with unsaturated fats and reduced consumption of ultra-processed products. While these patterns are linked to increased microbial diversity, increased capacity to produce short-chain fatty acids and reduced inflammatory tone, it is important to note that these patterns cannot be boiled down to one microbial signature [132,133]. Polyphenols are important due to variation in the conversion to bioactive metabolites by microbes during metabolism, but some of the polyphenols are poorly absorbed [134]. Fermented foods may include live microbes, microbial metabolites and bioactive peptides and their effects in the body will depend on the process and whether the microorganisms are alive and on diet consumption [135].

Physical activity provides a parallel mechanism to microbiota modulation in addition to the dietary pathway. There have been associations between exercise and microbial diversity, SCFA production, barrier function, and immune signaling; however, these effects are dependent on training status, the intensity of exercise, diet, and basal microbiota [83,136]. While the evidence of urolithin A in sleep-deprived mice is a plausible gut–muscle axis intervention, the data are not yet clinical and should not be generalized as an intervention for IBS or MASLD/MASH [137]. Physical activity should thus be considered a context-dependent microbiota modulator and not disease-specific microbiome therapy.

### 7.2. Prebiotics, Probiotics, Synbiotics, and Postbiotics

Prebiotics, probiotics, synbiotics and postbiotics are each a different class of interventions and should not be viewed as one and the same “microbiome product”. Prebiotics are used substrates that have the potential to improve the functions of host resident microbes, while probiotics are live microorganisms that need to be present in adequate quantities, viable, and of the correct strain to demonstrate the health claim [138,139]. Synbiotics involve the use of live microbes in combination with substrates that are selectively used, but the rationale needs to clarify whether the substrate promotes the growth of the administered strain or of the resident microbiota [138]. The differences with postbiotics are that they are inanimate microorganisms, with or without their components or metabolites; this means that they cannot be assessed according to the principles of colonization or viability of probiotics [140].

Clinical interpretation should be indication-specific. Pediatric gastroenterology guidance notes that the recommendations should be specific to the strain or strain combination, indication and dose and not general to the use of probiotics [139]. This is important because the efficacy of antibiotic-associated diarrhea, pouchitis, ulcerative colitis, or irritable bowel syndrome cannot be extrapolated from one product to another or from one disease to another. The influence of host factors in response is also complex. However, the colonization process, production of metabolites and clinical outcome may be influenced by baseline microbiota, diet, exposure to medications, and immune status and metabolic phenotype, making it difficult to universally affirm the beneficial effects of probiotics [141].

Use of prebiotics and synbiotics is at its best when combined with defined substrate use, SCFA production, barrier support or immune signaling, and tolerance and efficacy are dependent on dose, fermentability, and resident microbial capacity [142]. Consistency of manufacturing, source of the microbial population, process of microbial inactivation, dose, and clinical endpoint are still critical factors in reducing risks associated with the administration of live organisms to susceptible hosts; however, postbiotics may play a role in doing so [140,143]. Probiotic treatments that include multiple strains, rather than a single strain, often provide broader and more consistent beneficial effects across different health conditions because each strain can contribute distinct mechanisms of action [144,145,146]. While a single strain may target a specific pathway or imbalance, multi-strain formulations can work synergistically to enhance gut microbiota diversity, improve intestinal barrier function, modulate immune responses, and inhibit a wider range of pathogenic organisms [147]. Special care needs to be taken with multi-strain products. There is no correlation between number of strains and efficacy. There should be evidence for the formulation to be strain compatible and for dose contribution, target indication, and measured clinical benefit [139,141].

### 7.3. Fecal Microbiota Transplantation

FMT is most effective in recurrent CDI as this is a clearly identified failure of the ecology, in this case, antibiotic injury that allows *C. difficile* to flourish. FMT works more towards reestablishing community functions such as pathogen exclusion, nutrient competition, and bile acid conversion, instead of introducing diversity [98]. This is suggestive of AGA-specific logic supporting fecal microbiota-based therapy (FMT) for selected adults with recurrent CDI following standard antibiotic therapy, and the FDA’s approval of Vowst for preventing recurrent CDI after antibacterial treatment [99,100].

The evidence outside recurrent CDI is weaker as the therapeutic target is less well-defined. In IBD and IBS, guidelines are still conservative due to the diversity of disease mechanisms and the lack of long-term effects, responder profile, and safety profile of conventional FMT [99]. Microbiome and metabolic effects have been proposed in MASLD/MASH and metabolic disease, but are only suggested in small, randomized studies and may not be generalizable due to limited sample sizes, selection of donors, and short follow-up periods [110,148]. For autism and neurodegenerative diseases, there are no controlled clinical trials, the endpoints are inconsistent, and the reports are hypothesis generating [149,150].

Translation depends on variables that the term FMT can obscure. The effect of donor variability, stool screening, route of administration, dose, preparation method, recipient baseline microbiota, antibiotic pre-treatment, and engraftment durability can all impact the response [151,152]. Safety should not be an afterthought, as the transmission of multidrug-resistant organisms and enteric pathogens has resulted in FDA safety communications and tightened donor testing requirements [100]. There is also a current transition from informal donor stool collection to the use of regulated live biotherapeutic products (LBPs), which, although used to promote consistency, will present other questions regarding the manufacturing of these products, their potency, and post-treatment monitoring [149]. FMT should be considered a standard-of-care treatment for recurrent CDI. For other diseases, it is context dependent and investigational.

**Figure 2:**
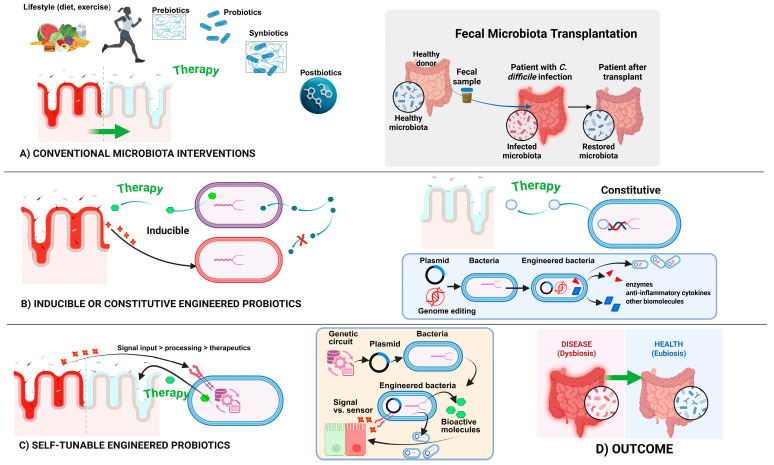
Conventional and engineered microbiota targeted intervention strategies. Panel (**A**) summarizes conventional approaches, including diet, physical activity, prebiotics, probiotics, synbiotics, postbiotics, and fecal microbiota transplantation. These approaches primarily modify microbial ecology, substrate availability, metabolite production, or colonization resistance. Panels (**B**,**C**) illustrate engineered microbial systems designed for constitutive, inducible, or self-tunable activity through genetic circuits, biosensors, therapeutic molecule delivery, enzyme production, anti-inflammatory signaling, or local metabolic modulation. Panel (**D**) should be interpreted conceptually. It does not imply that microbiota targeted interventions consistently or directly restore eubiosis, nor that engineered systems are clinically equivalent to established therapies. Clinical relevance depends on indication, evidence quality, safety, durability, host context, regulatory oversight, and post-treatment monitoring.

In Figure 2, conventional and engineered microbiota-targeted interventions are mapped onto a similar conceptual landscape. The conventional strategies primarily operate by changing the ecology broadly, such as by altering the availability of food or producing metabolites or by changing the colonization resistance or barriers. Engineered microbial system: more conditionally functional, such as for detecting signals of disease, delivering therapeutic molecules, and local metabolic modulation. The figure should be interpreted as a conceptual comparison between intervention strategies and not as proof of equal clinical maturity of all strategies, also noting that each is reliable in achieving the change from dysbiosis to eubiosis.

## 8. Precision Genetic Engineering and Engineered Microbial Therapeutics

### 8.1. Rationale for Engineered Microbial Therapeutics

The use of conventional microbiota modulation is clinically beneficial but not very precise. Diet, prebiotics, probiotics and FMT may be able to change the microbiota, but the effect of these interventions will vary based on the host microbiota, colonization resistance, substrate availability, host immunity and any medications being taken [153]. A probiotic strain can be ineffective for engraftment, exert different metabolites in different hosts, and/or only be effective under certain luminal conditions [154]. There is more evidence supporting FMT in recurrent CDI, but, outside of this context, controls for donor variability, functions transferred, durability and safety are still challenging [139]. In traditional interventions, these restrictions render the interventions broad ecologic interventions instead of narrowly-targeted therapeutics [21].

The goal of engineered microbial therapeutics is to address another issue. Engineered microbes are capable of not only passive colonization or passive change in community composition, but can also be engineered to respond to specific signals in disease settings, to produce specific molecules, to consume toxic substances, or to alter metabolic pathways. Microbial chassis, inducible promoters, and genetic circuits and containment strategies have been employed to design living sensors and local drug delivery systems with synthetic biology platforms [155,156]. The appeal is the mechanism (specification). The challenge is to keep the products of living therapeutics stable, controllable and safe within complex human ecosystems, before it can be argued that they should be used in clinical practice [157].

### 8.2. Engineered Probiotics and Microbial Chassis

The choice of microbial chassis should not be considered neutral, but a design choice. It is desirable that *Escherichia coli* Nissle 1917 should be genetically tractable and human exposed, but it has a facultative anaerobic biology which may not support the functions of strict gut anaerobes [21]. Lactococcus lactis is familiar in the food industry and has the potential of mucosal delivery but may not persist long enough for optimal therapy, which is desirable for safety reasons. The Bacteroides species are closer to the colon; however, they are difficult to develop because of the need to handle and contain the bacteria anaerobically [156]. Saccharomyces chassis can resist the acidity of the stomach and the effects of antibiotics better than some bacteria, but there are other immunological and manufacturing challenges [158].

Whether the engineered output will be helpful or harmful in therapy is dependent upon the therapeutic value of the chassis. The best delivery of enzymes is possible when the substrate to be attacked is known, such as in the case of metabolic detoxification or local change in metabolites [155]. Conceptually relevant to intestinal inflammation are anti-inflammatory cytokines, which can be thought of as having anti-inflammatory properties in general but which would be dangerous to use generally without spatial and dosage control [159]. Similarly, the production of barrier-supporting molecules, antimicrobial peptides, and metabolic pathway modulators is only meaningful if they are produced in the right place and at the right concentration [21]. The chassis fitness, genetic stability, therapeutic payload, containment and measurable host response under clinically relevant conditions and reproducible manufacturing constraints for engineered probiotics should therefore be considered, rather than their precision or novelty.

### 8.3. Smart Microbial Circuits and Disease-Responsive Systems

Smart microbial circuits shift the therapeutic claim from colonization to conditional action. Diseases may be present in a plant although no symptoms are observed, and a constitutive producer can release a payload when the disease is not active, reducing the safety and metabolic resources of the plant. In contrast, inducible systems are more defensible with output dependent on known inputs (such as metabolites of inflammation, oxygen tension, pH, quorum signals or any supplied molecules externally). The clinical utility of a biosensor depends on the specificity of the biosensor response, its stability and the ability to detect the signal, as well as the association with a clinically relevant decision [160].

In intestinal disease, the interest of inflammation-responsive circuits lies in the possibility of targeting them to the organ tissue that is inflamed, such as by means of tetrathionate, thiosulfate, nitric oxide or reactive oxygen species [161]. The problem is that the limit is the specificity. Circuit activation does not mean that an infection, injury or treatment-related inflammation has occurred [162], as these signals can be produced in a variety of situations. Quorum sensing systems can be used to regulate the behavior of the population as a whole (such as synchronized payload release or consortia cooperation) but as long as there is only poor or uneven engraftment in the intestinal niches, density-dependent systems can break down. Conceptually stronger self-tunable systems add the complexity of designing for the sensing, computing, and response components and the uncertainty of regulation [156]. Evaluation of the disease-responsive therapeutics should be done in addition to circuit function. The critical issues are the existence of input in patients, the proportionality of the output, the stability of the circuit and reliable shutdown or containment [157].

### 8.4. Limits of Current Evidence

Many engineered microbial therapeutics are still in the translational prototype stage, rather than being readily available in the clinic. Human ecosystems introduce variables which are hard to replicate in preclinical models, but can be demonstrated in these models under controlled conditions for sensing and payload delivery or metabolic conversion. The issue of colonization stability is at the heart of the issue. A strain capable of performing in vitro may not thrive, may limit its distribution to an area other than the intended niche, or may be outcompeted by the community of microbes that colonize it, dietary changes, antibiotics or inflammation [21,163].

There is a limitation of predictability when there is a host context. Baseline microbiota can influence substrate availability and ecological competition and cross-feeding, but host immunity can cause persistence shifts or trigger responses against the engineered organism or its payload [164]. Strain identity, genome stability, antimicrobial resistance, virulence potential, immune reactivity and manufacturing consistency are important safety assessment factors that need to be included [157,165]. There is low therapeutic output. Genetic circuits can lose function, undergo mutations, become metabolically burdensome or be expressed at levels that are too low, too high or too early or late for the biology of a disease [156].

The clinical issues relating to containment and reversibility are still not answered. Although the use of kill switches, auxotrophy and externally inducible systems makes for a safer approach, it is still important to note that there is always the risk of horizontal gene transfer, environmental release, uncontrolled persistence and inadequate shutdown in heterologous hosts [166]. This tension is illustrated by the fact that engineered probiotics for the treatment of inflammatory bowel disease (IBD) are possible both of delivering therapeutic anti-inflammatory effects or barrier-supportive products; however, substantial challenges of genetic modification of safety (GMO) and regulatory issues, along with clinical validation, remain. These should thus be introduced as experimental platforms, rather than interventions for clinical routine.

## 9. Translational Challenges, Safety, Regulation, and Future Directions

The biggest translation gap in microbiome science is the transition from association to inferences about therapeutic implications. Any difference between cases and controls in the microbiological feature could be due to the disease itself, diet, treatment, geographic location, genetic differences in the host population, sampling technique or analytical pipeline [167]. It is not alone a path to discover a cause or a therapeutic goal. While consensus recently has been called for using longitudinal designs, controlled perturbation, mechanistic coherence, and the use of the right preclinical models, the study of causality does not necessarily demand repeated cross-sectional association [18]. Reproducibility thus becomes more than an afterthought of a technical nature. Another issue is that stool samples, sequencing depth, extraction method, bioinformatics, clinical metadata and statistical adjustment all vary across cohorts, leading to changes in microbiome signatures. Another challenge is that the sampling and sequencing of stool, the depth of sequencing, the extraction method, the bioinformatics, clinical data and statistical adjustment differ across studies, leading to variations in microbiome signatures. This is exacerbated by small sample sizes, restricts the ability to perform subgroup analyses and raises the risk of false discovery. When there are unequal numbers of medications (such as antibiotics, metformin, bowel prep, and immunosuppressants) between comparison groups, underpowered studies can also be hard to interpret and make it difficult to distinguish the effects of the disease from the effects of the medicine.

Interindividual variation also weakens broad therapeutic claims. The composition and function of the microbiota may be influenced by diet, age, geographic location, antibiotics, proton pump inhibitors, metformin, bowel preparation, immune status and host genetics [168]. For clinical translation, trials that establish baseline microbiota, diet, medication exposure, disease phenotype and clinically meaningful endpoints are required to draw benefit to the microbiota modulation [169]. The trial design must also have sufficient follow-up, prespecified microbial endpoints, validated clinical endpoints and a plan for non-responders as there may be a lack of correlation between short-term compositional change and durable benefit. The same issues that plague diet microbiome research apply to studies of adherence: difficulty in measuring diet, short interventions, heterogeneity of adherence data, and heterogeneity of outcome measures, which makes results difficult to compare [170]. While animal and in vitro models will continue to be useful in mechanism, human studies will not replace animal studies due to differences in microbial flora, immune systems, diet, and dosing conditions between these systems [18].

Engineered microbial therapeutics are another type of risk. The colonization stability, spatial localization, durability of the circuit, payload expression and compatibility with the resident microbes are important factors of therapeutic performance [171]. The niche in a controlled model might not be the same niche found in patients, due to changes in niche by inflammation, diet, antibiotics, competition with microbes or the immune system [172]. There were no engineered therapeutic bacteria that were approved by 2024, which was mainly due to the need to demonstrate efficacy in combination with biosafety and containment [173]. Genome stability, virulence potential, antimicrobial resistance, immune reactivity, horizontal gene transfer, shedding, environmental release and manufacturing consistency are safety issues to be addressed. The relevance of the safety literature of probiotics is that commercially available microbes might harbor the genes for antimicrobial resistances and the gut resistome may facilitate horizontal transfer in a permissive ecological context [165,174,175].

Containment and reversibility are not known. Although these kill switches, auxotrophy, inducible circuits, encapsulation and systems which are controlled externally can minimize risk, they do not prevent the necessity of long-term monitoring of persistence, genetic drift, adverse events and ecological effects following treatment [157,166]. The product identity is not the only element that requires regulatory assessment—potency, dose, release control, off target activity, environmental containment and post-treatment surveillance are also required. Conflict-of-interest transparency is also a crucial consideration in an industry where products under the umbrella of probiotics and live biotherapeutics go hand in hand with scientific claims. The standards for medical publications call for disclosure of relationships which might bias, or seem to bias, interpretation [169]. Disciplined claims, enhanced clinical trials in humans, disease- and population-specific reporting standards, and clear identification and separation of evidence and commercial positioning will drive future progress.

## 10. Conclusions

The gut microbiota is a plausible therapeutic target, as it is involved in the maintenance of the epithelial barrier, production of microbial metabolites, transformation of bile acids, immune regulation, and communication between the gut and distant organs. However, while biological plausibility does not equate clinical certainty, it does imply that the link may be real. The microbiome results are not uniform and are highly dependent on the context, diet, geographical location, type of medications and other additives, host genetic makeup, stage of disease, sampling technique and analysis pipeline across metabolic, gastrointestinal, hepatic, cancer and neuroimmune diseases.

The good thing about the traditional treatments is that they are something that doctors are familiar with. Modulation of microbial ecology through diet, physical activity, prebiotics, probiotics, synbiotics, postbiotics and FM transplantations can have multiple, host-specific mechanisms of action. FMT has been the most obvious clinical case; in recurrent *C. difficile* infection, restoration of colonization resistance is a well-defined therapeutic goal.

Engineered microbial therapeutics have the advantage of being more precisely engineered in experiments, by having a sensor that can detect signals, a molecule that can be delivered or a metabolic alteration that can be done locally. Limitations to their clinical usefulness are colonization control, genetic stability, immune response, containment, reversibility, regulation, and long-term monitoring. More human trials and standardization, mechanistic validation, transparent reporting and careful regulation are needed to move forward.

## Figures and Tables

**Table 1:** Approximate microbial abundance and representative microbial groups across selected body and gastrointestinal sites [24,25,26].

Site	Concentration (CFU/mL)	pH	pO_2_ (~mmHg)	Main Microbial Populations
Oral cavity	10^11^–10^12^	6.2–7.5	83–113	*Corynebacteria* spp., *Lactobacillus*, *Streptococcus*
Stomach	10^7^	1.5–3.0	60–77	*H. pylori*, *Lactobacillus*, *Streptococcus*
Duodenum	10^7^	5.6–8.0	30	*Lactobacillus*, *Streptococcus*
Jejunum	10^7^	5.7–7.5	10–34	*Lactobacillus*, *Streptococcus*
Ileum	10^11^	5.7–7.5	10–34	*Lactobacillus*, *Streptococcus*
Colon	10^14^	6.7–8.5	0.5–11	*Bacteroides*, *Bifidobacterium* spp., *Clostridium* spp., *Enterococcus*, *E. coli*, *Enterococcus faecalis*, *Lactobacillus*
Skin	10^11^	5.0–5.5	145	*Actinobacteria*, *Bacteroidetes*, *Cyanobacteria* spp., *Firmicutes*, *Proteobacteria*

Abbreviations: CFU, colony-forming units; pO_2_, partial pressure of oxygen. Values are approximate and vary according to sampling method, diet, age, medication exposure, oxygen tension, pH, transit time, and disease state. Listed microbial groups are representative examples and should not be interpreted as exhaustive or fixed site-specific profiles.

## Data Availability

Data sharing not applicable to this article as no datasets were generated or analyzed during the current study.

## References

[1] Meldrum O.W., Yakubov G.E. (2025). Journey of dietary fiber along the gastrointestinal tract: Role of physical interactions, mucus, and biochemical transformations. Crit. Rev. Food Sci. Nutr..

[2] Ludwig K., Santoro L., Ingravallo G., Cazzato G., Giacometti C., Dall’Igna P. (2022). Congenital anomalies of the gastrointestinal tract: The liver, extrahepatic biliary tree and pancreas. Pathologica.

[3] Shalon D., Culver R.N., Grembi J.A., Folz J., Treit P.V., Shi H., Rosenberger F.A., Dethlefsen L., Meng X., Yaffe E. (2023). Profiling the human intestinal environment under physiological conditions. Nature.

[4] Yang K., Li G., Li Q., Wang W., Zhao X., Shao N., Qiu H., Liu J., Xu L., Zhao J. (2025). Distribution of gut microbiota across intestinal segments and their impact on human physiological and pathological processes. Cell Biosci..

[5] Khalil M., Di Ciaula A., Mahdi L., Jaber N., Di Palo D.M., Graziani A., Baffy G., Portincasa P. (2024). Unraveling the role of the human gut microbiome in health and diseases. Microorganisms.

[6] Matijašić M., Meštrović T., Čipčić Paljetak H., Perić M., Barešić A., Verbanac D. (2020). Gut microbiota beyond bacteria—Mycobiome, virome, archaeome, and eukaryotic parasites in IBD. Int. J. Mol. Sci..

[7] Leonard J.M., Del Toro D. (2023). Defining the Microbiome Components (Bacteria, Viruses, Fungi) and Microbiome Geodiversity. Surg. Infect..

[8] García-Bonete M.J., Rajan A., Suriano F., Layunta E. (2023). The Underrated Gut Microbiota Helminths, Bacteriophages, Fungi, and Archaea. Life.

[9] Hu C., Shen H. (2024). Microbes in Health and Disease: Human Gut Microbiota. Appl. Sci..

[10] Ma Z., Zuo T., Frey N., Rangrez A.Y. (2024). A Systematic Framework for Understanding the Microbiome in Human Health and Disease: From Basic Principles to Clinical Translation. Signal Transduct. Target. Ther..

[11] Safarchi A., Al-Qadami G., Tran C.D., Conlon M. (2025). Understanding Dysbiosis and Resilience in the Human Gut Microbiome: Biomarkers, Interventions, and Challenges. Front. Microbiol..

[12] Fu Y., Lyu J., Wang S. (2023). The Role of Intestinal Microbes on Intestinal Barrier Function and Host Immunity. Front. Immunol..

[13] Ney L.M., Wipplinger M., Grossmann M., Engert N., Wegner V.D., Mosig A.S. (2023). Short Chain Fatty Acids: Key Regulators of the Local and Systemic Immune Response in Inflammatory Diseases and Infections. Open Biol..

[14] Fogelson K.A., Dorrestein P.C., Zarrinpar A., Knight R. (2023). The Gut Microbial Bile Acid Modulation and Its Relevance to Digestive Health and Diseases. Gastroenterology.

[15] Di Ciaula A., Bonfrate L., Khalil M., Portincasa P. (2023). The interaction of bile acids and gut inflammation influences the pathogenesis of inflammatory bowel disease. Intern. Emerg. Med..

[16] Portincasa P., Bonfrate L., Vacca M., De Angelis M., Farella I., Lanza E., Khalil M., Wang D.Q., Sperandio M., Di Ciaula A. (2022). Gut microbiota and short chain fatty acids: Implications in glucose homeostasis. Int. J. Mol. Sci..

[17] Wensel C.R., Pluznick J.L., Salzberg S.L., Sears C.L. (2022). Next-Generation Sequencing: Insights to Advance Clinical Investigations of the Microbiome. J. Clin. Investig..

[18] Metwaly A., Kriaa A., Hassani Z., Carraturo F., Druart C., Pinto F., Asnicar F., Segata N., Morozova V., Sarathi A. (2025). A Consensus Statement on Establishing Causality, Therapeutic Applications and the Use of Preclinical Models in Microbiome Research. Nat. Rev. Gastroenterol. Hepatol..

[19] Portincasa P., Khalil M., Graziani A., Fruehbeck G., Baffy G., Garruti G., Di Ciaula A., Bonfrate L. (2024). Gut microbes in metabolic disturbances. Promising role for therapeutic manipulations?. Eur. J. Intern. Med..

[20] Rinella M.E., Lazarus J.V., Ratziu V., Francque S.M., Sanyal A.J., Kanwal F., Romero D., Abdelmalek M.F., Anstee Q.M., Arab J.P. (2023). A Multisociety Delphi Consensus Statement on New Fatty Liver Disease Nomenclature. J. Hepatol..

[21] Murali S.K., Mansell T.J. (2024). Next Generation Probiotics: Engineering Live Biotherapeutics. Biotechnol. Adv..

[22] Aggarwal N., Kitano S., Puah G.R.Y., Kittelmann S., Hwang I.Y., Chang M.W. (2023). Microbiome and Human Health: Current Understanding, Engineering, and Enabling Technologies. Chem. Rev..

[23] Winter S.E., Bäumler A.J. (2023). Gut Dysbiosis: Ecological Causes and Causative Effects on Human Disease. Proc. Natl. Acad. Sci. USA.

[24] Obayomi O., Olaniran A., Malomo A., Agaja F., Olojede A., Osemwegie O. (2025). Gut Dysbiosis and Chronic Diseases: Unraveling Contributing Factors, Health Implications, and Therapeutic Interventions. Food Humanit..

[25] de Vos W.M., Tilg H., Van Hul M., Cani P.D. (2022). Gut Microbiome and Health: Mechanistic Insights. Gut.

[26] Hou K., Wu Z.-X., Chen X.-Y., Wang J.-Q., Zhang D., Xiao C., Zhu D., Koya J.B., Wei L., Li J. (2022). Microbiota in Health and Diseases. Signal Transduct. Target. Ther..

[27] Alagiakrishnan K., Morgadinho J., Halverson T. (2024). Approach to the diagnosis and management of dysbiosis. Front. Nutr..

[28] Neurath M.F., Artis D., Becker C. (2025). The Intestinal Barrier: A Pivotal Role in Health, Inflammation, and Cancer. Lancet Gastroenterol. Hepatol..

[29] Cusumano G., Flores G.A., Venanzoni R., Angelini P. (2025). The impact of antibiotic therapy on intestinal microbiota: Dysbiosis, antibiotic resistance, and restoration strategies. Antibiotics.

[30] Yu S., Sun Y., Shao X., Zhou Y., Yu Y., Kuai X., Zhou C. (2022). Leaky gut in IBD: Intestinal barrier–gut microbiota interaction. J. Microbiol. Biotechnol..

[31] Gieryńska M., Szulc-Dąbrowska L., Struzik J., Mielcarska M.B., Gregorczyk-Zboroch K.P. (2022). Integrity of the intestinal barrier: The involvement of epithelial cells and microbiota—A mutual relationship. Animals.

[32] Di Vincenzo F., Del Gaudio A., Petito V., Lopetuso L.R., Scaldaferri F. (2024). Gut Microbiota, Intestinal Permeability, and Systemic Inflammation: A Narrative Review. Intern. Emerg. Med..

[33] Blachier F. (2023). Amino acid-derived bacterial metabolites in the colorectal luminal fluid: Effects on microbial communication, metabolism, physiology, and growth. Microorganisms.

[34] Ali Q., Ma S., La S., Guo Z., Liu B., Gao Z., Farooq U., Wang Z., Zhu X., Cui Y. (2022). Microbial short-chain fatty acids: A bridge between dietary fibers and poultry gut health—A review. Anim. Biosci..

[35] Zhang D., Jian Y.P., Zhang Y.N., Li Y., Gu L.T., Sun H.H., Liu M.D., Zhou H.L., Wang Y.S., Xu Z.X. (2023). Short-Chain Fatty Acids in Diseases. Cell Commun. Signal..

[36] Mohanty I., Allaband C., Mannochio-Russo H., El Abiead Y., Hagey L.R., Knight R., Dorrestein P.C. (2024). The changing metabolic landscape of bile acids–keys to metabolism and immune regulation. Nat. Rev. Gastroenterol. Hepatol..

[37] Režen T., Rozman D., Kovács T., Kovács P., Sipos A., Bai P., Mikó E. (2022). The role of bile acids in carcinogenesis. Cell. Mol. Life Sci..

[38] Collins S.L., Stine J.G., Bisanz J.E., Okafor C.D., Patterson A.D. (2023). Bile Acids and the Gut Microbiota: Metabolic Interactions and Impacts on Disease. Nat. Rev. Microbiol..

[39] Macpherson A.J., Pachnis V., Prinz M. (2023). Boundaries and Integration between Microbiota, the Nervous System, and Immunity. Immunity.

[40] Liu C., Sun M., Zhao Z., Yang Y., Yang Y., Zhao Y., Zhang R., Du X., Liu X., Ran S. (2026). Urolithin A: A multi-target therapeutic candidate derived from the gut microbiota for obesity and metabolic dysfunction. Front. Endocrinol..

[41] Campbell C., Kandalgaonkar M.R., Golonka R.M., Yeoh B.S., Vijay-Kumar M., Saha P. (2023). Crosstalk between Gut Microbiota and Host Immunity: Impact on Inflammation and Immunotherapy. Biomedicines.

[42] Lee H.K., Kim N.E., Shin C.M., Oh T.J., Yoon H., Park Y.S., Kim N., Won S., Lee D.H. (2024). Gut Microbiome Signature of Metabolically Healthy Obese Individuals According to Anthropometric, Metabolic and Inflammatory Parameters. Sci. Rep..

[43] Nóbrega R., Costa C.F., Cerqueira Ó., Inês A., Carrola J.S., Gonçalves C. (2025). Association between gut microbiota and pediatric obesity: A systematic review. Nutrition.

[44] Zhang K., Zhang Q., Qiu H., Ma Y., Hou N., Zhang J., Kan C., Han F., Sun X., Shi J. (2024). The Complex Link between the Gut Microbiome and Obesity Associated Metabolic Disorders: Mechanisms and Therapeutic Opportunities. Heliyon.

[45] Park S., Zhang T., Yue Y., Wu X. (2022). Effects of bile acid modulation by dietary fat, cholecystectomy, and bile acid sequestrant on energy, glucose, and lipid metabolism and gut microbiota in mice. Int. J. Mol. Sci..

[46] Pedroza Matute S., Iyavoo S. (2023). Exploring the gut microbiota: Lifestyle choices, disease associations, and personal genomics. Front. Nutr..

[47] Fan Y., Liu Y., Shao C., Jiang C., Wu L., Xiao J., Tang L. (2025). Gut microbiota-targeted therapeutics for metabolic disorders: Mechanistic insights into the synergy of probiotic-fermented herbal bioactives. Int. J. Mol. Sci..

[48] Chen K., Wang H., Yang X., Tang C., Hu G., Gao Z. (2024). Targeting Gut Microbiota as a Therapeutic Target in T2DM: A Review of Multi Target Interactions of Probiotics, Prebiotics, Postbiotics, and Synbiotics with the Intestinal Barrier. Pharmacol. Res..

[49] Yu Y., Ding Y., Wang S., Jiang L. (2025). Gut Microbiota Dysbiosis and Its Impact on Type 2 Diabetes: From Pathogenesis to Therapeutic Strategies. Metabolites.

[50] Chong S., Lin M., Chong D., Jensen S., Lau N.S. (2025). A Systematic Review on Gut Microbiota in Type 2 Diabetes Mellitus. Front. Endocrinol..

[51] Bednarska N.G., Håberg A.K. (2025). Understanding Patterns of the Gut Microbiome May Contribute to the Early Detection and Prevention of Type 2 Diabetes Mellitus: A Systematic Review. Microorganisms.

[52] Letchumanan G., Abdullah N., Marlini M., Baharom N., Lawley B., Omar M.R., Mohideen F.B., Addnan F.H., Nur Fariha M.M., Ismail Z. (2022). Gut microbiota composition in prediabetes and newly diagnosed type 2 diabetes: A systematic review of observational studies. Front. Cell. Infect. Microbiol..

[53] Glaros S.B., Mishra S.P., Jain S., Davis F.S., Gabel S.A., Mueller G.A., Jarmusch A.K., Mabundo L., Courville A.B., Walter M.F. (2025). Systemic and Gut Microbiome Changes with Metformin and Liraglutide in Youth Onset Type 2 Diabetes: The MIGHTY Study. Gut Microbes.

[54] Yang T., Maki K.A., Marques F.Z., Cai J., Joe B., Pepine C.J., Pluznick J.L., Meyer K.A., Kirabo A., Bennett B.J. (2025). Hypertension and the Gut Microbiome: A Science Advisory from the American Heart Association. Hypertension.

[55] Tortelote G.G. (2026). Therapeutic strategies for hypertension: Exploring the role of microbiota-derived short-chain fatty acids in kidney physiology and development. Pediatr. Nephrol..

[56] Zhou J., Zhang H., Wang Y., Yang L., Liu A., Chen G., Tao F., Liu K., Zhang D. (2024). The association between circulating short-chain fatty acids and blood pressure in Chinese elderly population. Sci. Rep..

[57] Zhen J., Zhou Z., He M., Han H.X., Lv E.H., Wen P.B., Liu X., Wang Y.T., Cai X.C., Tian J.Q. (2023). The Gut Microbial Metabolite Trimethylamine N Oxide and Cardiovascular Diseases. Front. Endocrinol..

[58] Shremo Msdi A., Haghparast A., Garey K.W., Wang E.M. (2025). Microbiome-Based Therapeutics for Salt-Sensitive Hypertension: A Scoping Review. Nutrients.

[59] Wang L., Hu J. (2024). Unraveling the Gut Microbiota’s Role in Salt-Sensitive Hypertension: Current Evidences and Future Directions. Front. Cardiovasc. Med..

[60] Shan Y., Lee M., Chang E.B. (2022). The gut microbiome and inflammatory bowel diseases. Annu. Rev. Med..

[61] Świrkosz G., Szczygieł A., Logoń K., Wrześniewska M., Gomułka K. (2023). The role of the microbiome in the pathogenesis and treatment of ulcerative colitis—A literature review. Biomedicines.

[62] Jauregui Amezaga A., Smet A. (2024). The Microbiome in Inflammatory Bowel Disease. J. Clin. Med..

[63] Xie H., Yu S., Tang M., Xun Y., Shen Q., Wu G. (2025). Gut Microbiota Dysbiosis in Inflammatory Bowel Disease: Interaction with Intestinal Barriers and Microbiota Targeted Treatment Options. Front. Cell. Infect. Microbiol..

[64] Cabezas Cruz A., Bermúdez Humarán L.G. (2024). Exploring the Relationship between *Faecalibacterium duncaniae* and *Escherichia coli* in Inflammatory Bowel Disease (IBD): Insights and Implications. Comput. Struct. Biotechnol. J..

[65] Ambat A., Antony L., Maji A., Ghimire S., Mattiello S., Kashyap P.C., More S., Sebastian V., Scaria J. (2024). Enhancing recovery from gut microbiome dysbiosis and alleviating DSS-induced colitis in mice with a consortium of rare short-chain fatty acid-producing bacteria. Gut Microbes.

[66] Zheng L., Duan S.L., Dai Y.C., Wu S.C. (2022). Role of adherent invasive *Escherichia coli* in pathogenesis of inflammatory bowel disease. World J. Clin. Cases.

[67] Ma Z., Shi H., Bai X., Wang Z., Cao J., Dong Y., Chen Y. (2026). Host metabolism shapes the intestinal microbiota: A top-down paradigm of environmental selection pressure. Gut Microbes.

[68] Stolfi C., Maresca C., Monteleone G., Laudisi F. (2022). Implication of intestinal barrier dysfunction in gut dysbiosis and diseases. Biomedicines.

[69] Johnstone K.F., Herzberg M.C. (2022). Antimicrobial peptides: Defending the mucosal epithelial barrier. Front. Oral Health.

[70] Hu Y., Chen Z., Xu C., Kan S., Chen D. (2022). Disturbances of the gut microbiota and microbiota-derived metabolites in inflammatory bowel disease. Nutrients.

[71] Mukherjee A., Breselge S., Dimidi E., Marco M.L., Cotter P.D. (2024). Fermented foods and gastrointestinal health: Underlying mechanisms. Nat. Rev. Gastroenterol. Hepatol..

[72] Bernardi F., Fanizzi F., Parigi T.L., Zilli A., Allocca M., Furfaro F., Peyrin-Biroulet L., Danese S., D’Amico F. (2024). Role of probiotics in the management of patients with ulcerative colitis and pouchitis. Microorganisms.

[73] Quiroga-Centeno A.C., Atanasova K., Ebert M.P., Thomann A.K., Reindl W. (2025). Emerging Microbiome Directed Therapies in Inflammatory Bowel Disease: Beyond Diet Modification and FMT. Semin. Immunopathol..

[74] Hillestad E.M., van der Meeren A., Nagaraja B.H., Bjørsvik B.R., Haleem N., Benitez-Paez A., Sanz Y.c., Hausken T., Lied G.A., Lundervold A. (2022). Gut bless you: The microbiota-gut-brain axis in irritable bowel syndrome. World J. Gastroenterol..

[75] Lupu V.V., Adam Raileanu A., Mihai C.M., Moraru E., Stoica I., Tarca E., Trandafir L.M. (2023). Emerging Role of the Gut Microbiome in Post Infectious Irritable Bowel Syndrome: A Literature Review. World J. Gastroenterol..

[76] Warren A., Nyavor Y., Zarabian N., Mahoney A., Frame L.A. (2024). The microbiota-gut-brain-immune interface in the pathogenesis of neuroinflammatory diseases: A narrative review of the emerging literature. Front. Immunol..

[77] Li X., Yuan Q., Huang H., Wang L. (2025). Gut Microbiota in Irritable Bowel Syndrome: A Narrative Review. Front. Immunol..

[78] Zhang X., Jin W.W., Wang H.G. (2024). Correlation between the neuroendocrine axis, microbial species, inflammatory response, and gastrointestinal symptoms in irritable bowel syndrome. World J. Gastroenterol..

[79] Zhou Y., Tao E. (2026). The role of intestinal gases in pediatric functional constipation: A narrative review of pathophysiology and emerging therapeutics. Front. Nutr..

[80] Ju X., Jiang Z., Ma J., Yang D. (2024). Changes in Fecal Short Chain Fatty Acids in IBS Patients Compared with Healthy Controls: A Systematic Review and Meta Analysis. Nutrients.

[81] Deng M., Liu J., Zhang L., Lou Y., Qiu Y. (2024). Identification of molecular subtypes based on bile acid metabolism in cholangiocarcinoma. BMC Cancer.

[82] Min Y.W., Rezaie A., Pimentel M. (2022). Bile Acid and Gut Microbiota in Irritable Bowel Syndrome. J. Neurogastroenterol. Motil..

[83] Li C., Li J., Zhou Q., Wang C., Hu J., Liu C. (2024). Effects of Physical Exercise on the Microbiota in Irritable Bowel Syndrome. Nutrients.

[84] Rahmani S., Mas-Orea X., Chirdo F.G., Verdu E.F. (2026). Gene-environment interactions in celiac disease: The role of the intestinal epithelium. Lifestyle Genom..

[85] Patt Y.S., Lahat A., David P., Patt C., Eyade R., Sharif K. (2023). Unraveling the immunopathological landscape of celiac disease: A comprehensive review. Int. J. Mol. Sci..

[86] Stanciu D., Staykov H., Dragomanova S., Tancheva L., Pop R.S., Ielciu I., Crișan G. (2024). Gluten Unraveled: Latest Insights on Terminology, Diagnosis, Pathophysiology, Dietary Strategies, and Intestinal Microbiota Modulations—A Decade in Review. Nutrients.

[87] Luz V.C.C., Pereira S.G. (2024). Celiac Disease Gut Microbiome Studies in the Third Millennium: Reviewing the Findings and Gaps of Available Literature. Front. Med. Technol..

[88] Di Stasio L., Mamone G. (2025). Gluten Proteins: Beneficial Factors and Toxic Triggers in Human Health. Foods.

[89] Roque A., Pereira S.G. (2024). Bacteria: Potential Make or Break Determinants of Celiac Disease. Int. J. Mol. Sci..

[90] Matera M., Guandalini S. (2024). How the Microbiota May Affect Celiac Disease and What We Can Do. Nutrients.

[91] Valitutti F., Cavalli E., Leter B., Leonard M., Alessio F., Cucchiara S. (2025). Coeliac disease and microbiota: Is it time for personalised biotics intervention? A scoping review. BMJ Nutr. Prev. Health.

[92] Constante M., Libertucci J., Galipeau H.J., Szamosi J.C., Rueda G., Miranda P.M., Pinto-Sanchez M.I., Southward C.M., Rossi L., Fontes M.E. (2022). Biogeographic Variation and Functional Pathways of the Gut Microbiota in Celiac Disease. Gastroenterology.

[93] Annunziato A., Vacca M., Cristofori F., Dargenio V.N., Celano G., Francavilla R., De Angelis M. (2024). Celiac Disease: The Importance of Studying the Duodenal Mucosa-Associated Microbiota. Nutrients.

[94] Lupu V.V., Trandafir L.M., Raileanu A.A., Mihai C.M., Morariu I.D., Starcea I.M., Mocanu A., Butnariu L.I., Stoleriu G., Salaru D.L. (2023). Advances in understanding the human gut microbiota and its implication in pediatric celiac disease—A narrative review. Nutrients.

[95] Kolotylo V., Piwowarek K., Kieliszek M. (2023). Microbiological transglutaminase: Biotechnological application in the food industry. Open Life Sci..

[96] Parrotta L., Tanwar U.K., Aloisi I., Sobieszczuk-Nowicka E., Arasimowicz-Jelonek M., Del Duca S. (2022). Plant transglutaminases: New insights in biochemistry, genetics, and physiology. Cells.

[97] Spigaglia P. (2024). *Clostridioides difficile* and Gut Microbiota: From Colonization to Infection and Treatment. Pathogens.

[98] McMillan A.S., Theriot C.M. (2024). Bile Acids Impact the Microbiota, Host, and Clostridioides difficile Interactions. Gut Microbes.

[99] Peery A.F., Kelly C.R., Kao D., Vaughn B.P., Lebwohl B., Singh S., Imdad A., Altayar O. (2024). AGA Clinical Practice Guideline on Fecal Microbiota Based Therapies for Select Gastrointestinal Diseases. Gastroenterology.

[100] US Food and Drug Administration (2023). FDA Approves First Orally Administered Fecal Microbiota Product for the Prevention of Recurrence of *Clostridioides difficile* Infection. https://www.fda.gov/news-events/press-announcements/fda-approves-first-orally-administered-fecal-microbiota-product-prevention-recurrence-clostridioides.

[101] Tacke F., Horn P., Wong V.W.-S., Ratziu V., Bugianesi E., Francque S., Zelber-Sagi S., Valenti L., Roden M., Schick F. (2024). EASL–EASD–EASO Clinical Practice Guidelines on the Management of Metabolic Dysfunction-Associated Steatotic Liver Disease (MASLD). J. Hepatol..

[102] Jiang L., Du L.D., Zeng J., Chu H.K., Peng Z., Fan J.G. (2025). Pediatric metabolic dysfunction–associated steatotic liver disease and the gut microbiome: From research landscape to targeted modulation. Clin. Mol. Hepatol..

[103] Schnabl B., Damman C.J., Carr R.M. (2025). Metabolic Dysfunction-Associated Steatotic Liver Disease and the Gut Microbiome: Pathogenic Insights and Therapeutic Innovations. J. Clin. Investig..

[104] Wang L.J., Sun J.G., Chen S.C., Sun Y.L., Zheng Y., Feng J.C. (2025). The Role of Intestinal Flora in Metabolic Dysfunction-Associated Steatotic Liver Disease and Treatment Strategies. Front. Med..

[105] Cebi M., Yilmaz Y. (2025). Epithelial barrier hypothesis in the context of nutrition, microbial dysbiosis, and immune dysregulation in metabolic dysfunction-associated steatotic liver. Front. Immunol..

[106] Olaru-Stavila C., Steinmann S.M., Mester P., Müller M., Tcaciuc E., Gülow K. (2026). The Gastrointestinal Barrier—Mechanisms of Barrier Dysfunction in Liver Cirrhosis and Spontaneous Bacterial Peritonitis. Biomedicines.

[107] Rusman R.D., Akil F., Parewangi M.L., Daud N.M., Bachtiar R., Kusuma S.H., Rifai A. (2025). Gut microbiota and metabolic-associated steatosis liver disease: Unveiling mechanisms and opportunities for therapeutic intervention. World J. Exp. Med..

[108] Fleishman J.S., Kumar S. (2024). Bile acid metabolism and signaling in health and disease: Molecular mechanisms and therapeutic targets. Signal Transduct. Target. Ther..

[109] Stamation R. (2025). Endogenous ethanol production in the human alimentary tract: A literature review. J. Gastroenterol. Hepatol..

[110] Bahitham W., Banoun Y., Aljahdali M., Almuaiqly G., Bahshwan S.M., Aljahdali L., Sanai F.M., Rosado A.S., Sergi C.M. (2025). “Trust your gut”: Exploring the connection between gut microbiome dysbiosis and the advancement of Metabolic Associated Steatosis Liver Disease (MASLD)/Metabolic Associated Steatohepatitis (MASH): A systematic review of animal and human studies. Front. Nutr..

[111] Joo J.E., Chu Y.L., Georgeson P., Walker R., Mahmood K., Clendenning M., Meyers A.L., Como J., Joseland S., Preston S.G. (2024). Intratumoral Presence of the Genotoxic Gut Bacteria pks+ E. coli, Enterotoxigenic Bacteroides fragilis, and Fusobacterium nucleatum and Their Association with Clinicopathological and Molecular Features of Colorectal Cancer. Br. J. Cancer.

[112] Zepeda-Rivera M., Minot S.S., Bouzek H., Wu H., Blanco-Míguez A., Manghi P., Jones D.S., LaCourse K.D., Wu Y., McMahon E.F. (2024). A Distinct Fusobacterium nucleatum Clade Dominates the Colorectal Cancer Niche. Nature.

[113] Yang J., Wang X., Hu T., Huang H., Chen G., Jin B., Zeng G., Liu J. (2024). Entero-toxigenic Bacteroides fragilis contributes to intestinal barrier injury and colorectal cancer progression by mediating the BFT/STAT3/ZEB2 pathway. Cell Cycle.

[114] Luo Y.C., Huang X.T., Wang R., Lin Y.J., Sun J.X., Li K.F., Wang D.Y., Yan Y., Qiao Y.K. (2025). Advancements in understanding tumor-resident bacteria and their application in cancer therapy. Mil. Med. Res..

[115] Wekking D., Silva C.A., Viscò R., Denaro N., Lambertini M., Maccioni A., Loddo E., Willard-Gallo K., Scartozzi M., Derosa L. (2025). The interplay between gut microbiota, antibiotics, and immune checkpoint inhibitors in patients with cancer: A narrative review with biological and clinical aspects. Crit. Rev. Oncol./Hematol..

[116] Lu Y., Yuan H., Liang S., Li D., Jiang P., Wang X., Zhang K., Liu D. (2025). Microbial metabolite-driven immune reprogramming in tumor immunotherapy: Mechanisms and therapeutic perspectives. Front. Immunol..

[117] Tito R.Y., Verbandt S., Aguirre Vazquez M., Lahti L., Verspecht C., Lloréns-Rico V., Vieira-Silva S., Arts J., Falony G., Dekker E. (2024). Microbiome confounders and quantitative profiling challenge predicted microbial targets in colorectal cancer development. Nat. Med..

[118] Chen G., Ren Q., Zhong Z., Li Q., Huang Z., Zhang C., Yuan H., Feng Z., Chen B., Wang N. (2024). Exploring the gut microbiome’s role in colorectal cancer: Diagnostic and prognostic implications. Front. Immunol..

[119] Gazzaniga F.S., Kasper D.L. (2025). The gut microbiome and cancer response to immune checkpoint inhibitors. J. Clin. Investig..

[120] Puricelli C., Rolla R., Gigliotti L., Boggio E., Beltrami E., Dianzani U., Keller R. (2022). The gut-brain-immune axis in autism spectrum disorders: A state-of-art report. Front. Psychiatry.

[121] Menozzi E., Macnaughtan J., Schapira A.H. (2021). The gut-brain axis and Parkinson disease: Clinical and pathogenetic relevance. Ann. Med..

[122] Klann E.M., Dissanayake U., Gurrala A., Farrer M., Shukla A.W., Ramirez-Zamora A., Mai V., Vedam-Mai V. (2022). The gut–brain axis and its relation to Parkinson’s disease: A review. Front. Aging Neurosci..

[123] Travagli R.A., Browning K.N., Camilleri M. (2020). Parkinson disease and the gut: New insights into pathogenesis and clinical relevance. Nat. Rev. Gastroenterol. Hepatol..

[124] Yang R., Gao G., Yang H. (2022). The pathological mechanism between the intestine and brain in the early stage of Parkinson’s disease. Front. Aging Neurosci..

[125] Kalyanaraman B., Cheng G., Hardy M. (2024). Gut microbiome, short-chain fatty acids, alpha-synuclein, neuroinflammation, and ROS/RNS: Relevance to Parkinson’s disease and therapeutic implications. Redox Biol..

[126] Panaitescu P.Ș., Răzniceanu V., Mocrei-Rebrean Ș.M., Neculicioiu V.S., Dragoș H.M., Costache C., Filip G.A. (2024). The Effect of Gut Microbiota-Targeted Interventions on Neuroinflammation and Motor Function in Parkinson’s Disease Animal Models—A Systematic Review. Curr. Issues Mol. Biol..

[127] Morais L.H., Boktor J.C., MahmoudianDehkordi S., Kaddurah Daouk R., Mazmanian S.K. (2024). α-Synuclein Overexpression and the Microbiome Shape the Gut and Brain Metabolome in Mice. npj Park. Dis..

[128] West K.A., Yin X., Rutherford E.M., Wee B., Choi J., Chrisman B.S., Dunlap K.L., Hannibal R.L., Hartono W., Lin M. (2022). Multi Angle Meta Analysis of the Gut Microbiome in Autism Spectrum Disorder: A Step toward Understanding Patient Subgroups. Sci. Rep..

[129] Petropoulos A., Stavropoulou E., Tsigalou C., Bezirtzoglou E. (2025). Microbiota Gut Brain Axis and Autism Spectrum Disorder: Mechanisms and Therapeutic Perspectives. Nutrients.

[130] Sun W., Ma L., Feng X., Fan Y., Cai Y., Li X. (2025). Efficacy of gut microbiota-based therapy for autism Spectrum Disorder and attention Deficit Hyperactivity Disorder: A systematic review and meta-analysis. Psychol. Health Med..

[131] Facchin S., Bertin L., Bonazzi E., Lorenzon G., De Barba C., Barberio B., Zingone F., Maniero D., Scarpa M., Ruffolo C. (2024). Short-Chain Fatty Acids and Human Health: From Metabolic Pathways to Current Therapeutic Implications. Life.

[132] Perrone P., D’Angelo S. (2025). Gut Microbiota Modulation Through Mediterranean Diet Foods: Implications for Human Health. Nutrients.

[133] Khalil M., Abdallah H., Razuka-Ebela D., Calasso M., De Angelis M., Portincasa P. (2023). The impact of Za’atar antioxidant compounds on the gut microbiota and gastrointestinal disorders: Insights for future clinical applications. Antioxidants.

[134] Meiners F., Ortega-Matienzo A., Fuellen G., Barrantes I. (2025). Gut Microbiome Mediated Health Effects of Fiber and Polyphenol Rich Dietary Interventions. Front. Nutr..

[135] Valentino V., Magliulo R., Farsi D., Cotter P.D., O’Sullivan O., Ercolini D., De Filippis F. (2024). Fermented foods, their microbiome and its potential in boosting human health. Microb. Biotechnol..

[136] Varghese S., Rao S., Khattak A., Zamir F., Chaari A. (2024). Physical Exercise and the Gut Microbiome: A Bidirectional Relationship. Nutrients.

[137] Zhu H., Zhao H., Qian H., Liu C. (2024). Urolithin A Ameliorates Athletic Ability and Intestinal Microbiota in Sleep Deprivation from the Perspective of the Gut Muscle Axis. Mol. Nutr. Food Res..

[138] Al-Habsi N., Al-Khalili M., Haque S.A., Elias M., Olqi N.A., Al Uraimi T. (2024). Health Benefits of Prebiotics, Probiotics, Synbiotics, and Postbiotics. Nutrients.

[139] Hojsak I., Kolaček S. (2024). Role of Probiotics in the Treatment and Prevention of Common Gastrointestinal Diseases in Children. Pediatr. Gastroenterol. Hepatol. Nutr..

[140] Vinderola G., Sanders M.E., Cunningham M., Hill C. (2024). Frequently Asked Questions about the ISAPP Postbiotic Definition. Front. Microbiol..

[141] Manan M.A. (2025). The role of probiotics in personalized therapeutics: Advances in gut microbe-driven interventions. Microbe.

[142] Smolinska S., Popescu F.-D., Zemelka-Wiacek M. (2025). A Review of the Influence of Prebiotics, Probiotics, Synbiotics, and Postbiotics on the Human Gut Microbiome and Intestinal Integrity. J. Clin. Med..

[143] Amobonye A., Pillay B., Hlope F., Asong S.T., Pillai S. (2025). Postbiotics: An Insightful Review of the Latest Category in Functional Biotics. World J. Microbiol. Biotechnol..

[144] Mörkl S., Narrath M., Schlotmann D., Sallmutter M.T., Putz J., Lang J., Brandstätter A., Pilz R., Karl Lackner H., Goswami N. (2025). Multi-species probiotic supplement enhances vagal nerve function–results of a randomized controlled trial in patients with depression and healthy controls. Gut Microbes.

[145] Horvath A., Haller R., Schmid-Zalaudek K., Goaswami N., Wagner-Skacel J., Habisch H., Madl T., Stadlbauer V. (2026). The beneficial effect of a multispecies probiotic intervention on quality of sleep—A randomized, double-blinded, placebo-controlled study. J. Psychiatr. Res..

[146] Strauss M., Mičetić Turk D., Lorber M., Pogačar M.Š., Koželj A., Tušek Bunc K., Fijan S. (2023). The multi-strain probiotic OMNi-BiOTiC^®^ active reduces the duration of acute upper respiratory disease in older people: A double-blind, randomised, controlled clinical trial. Microorganisms.

[147] Kwoji I.D., Aiyegoro O.A., Okpeku M., Adeleke M.A. (2021). Multi-strain probiotics: Synergy among isolates enhances biological activities. Biology.

[148] Groenewegen B., Ruissen M.M., Crossette E., Menon R., Prince A.L., Norman J.M., Ballieux B.E.P.B., Lamb H.J., Terveer E.M., Keller J.J. (2025). Consecutive fecal microbiota transplantation for metabolic dysfunction-associated steatotic liver disease: A randomized controlled trial. Gut Microbes.

[149] Sahle Z., Engidaye G., Shenkute Gebreyes D., Adenew B., Abebe T.A. (2024). Fecal microbiota transplantation and next-generation therapies: A review on targeting dysbiosis in metabolic disorders and beyond. SAGE Open Med..

[150] Liber A., Więch M. (2025). The Impact of Fecal Microbiota Transplantation on Gastrointestinal and Behavioral Symptoms in Children and Adolescents with Autism Spectrum Disorder: A Systematic Review. Nutrients.

[151] Liu C.S., Merrick B., Taboun Z.S., Mullish B.H., Goldenberg S.D., Terveer E.M., Porcari S., Bradbury R.S., Ianiro G., Ng S.C. (2026). Towards optimising and standardising donor screening for faecal microbiota transplantion. Gut.

[152] Gopalakrishnan V., Dozier E.A., Glover M.S., Novick S., Ford M., Morehouse C., Warrener P., Caceres C., Hess S., Sellman B.R. (2021). Engraftment of bacteria after fecal microbiota transplantation is dependent on both frequency of dosing and duration of preparative antibiotic regimen. Microorganisms.

[153] Woodworth M.H., Conrad R.E., Haldopoulos M., Pouch S.M., Babiker A., Mehta A.K., Sitchenko K.L., Wang C.H., Strudwick A., Ingersoll J.M. (2023). Fecal microbiota transplantation promotes reduction of antimicrobial resistance by strain replacement. Sci. Transl. Med..

[154] Guggeis M.A., Andreani N.A., López-Agudelo V.A., Tran F., Kadibalban A.S., Moors K.A., Marinos G., Saboukh A., Harris D., Falk-Paulsen M. (2025). Cross-species engraftment biases and metabolic divergence in gnotobiotic mice humanized with ulcerative colitis microbiota. Gut Microbes.

[155] Huang Y., Lin X., Yu S., Chen R., Chen W. (2022). Intestinal Engineered Probiotics as Living Therapeutics. ACS Synth. Biol..

[156] Kim K., Kim S., Ryu C.M. (2023). Systems and Synthetic Biology Driven Engineering of Live Bacterial Therapeutics: Towards Advanced Microbiome Therapeutics. Front. Bioeng. Biotechnol..

[157] Selvakumar R., Kumar I., Onajobi G.J., Yu Y., Wilson C.J. (2024). Engineering living therapeutics and diagnostics: A new frontier in human health. Curr. Opin. Syst. Biol..

[158] Kwak S. (2024). Therapeutic Applications of Native and Engineered Saccharomyces Yeasts. Fermentation.

[159] Campos G.M., Américo M.F., Dos Santos Freitas A., Barroso F.A.L., Dutra J.C.F., Quaresma L.S., Cordeiro B.F., Laguna J.G., De Jesus L.C.L., Fontes A.M. (2024). *Lactococcus lactis* as an Interleukin Delivery System for Prophylaxis and Treatment of Inflammatory and Autoimmune Diseases. Probiotics Antimicrob. Proteins.

[160] Tanniche I., Behkam B. (2023). Engineered Live Bacteria as Disease Detection and Diagnosis Tools. J. Biol. Eng..

[161] Abot A., Fried S., Cani P.D., Knauf C. (2022). Reactive oxygen species/reactive nitrogen species as messengers in the gut: Impact on physiology and metabolic disorders. Antioxid. Redox Signal..

[162] Kong C., Huang L.-B., Yang M.-F., Yue N.-N., Luo D., Zhang Y., Tian C.-M., Song Y., Wei D.-R., Shi R.-Y. (2025). Microbiome engineering: Unlocking therapeutic potential in inflammatory bowel disease. Front. Microbiol..

[163] Zhang L., Chen N., Chen H., Tang C., Wang J., Wang Y., Zhang Y., Guo H., Yuan J. (2025). Recent advances of engineered probiotics for therapeutic applications. BioDesign Res..

[164] Bakkeren E., Piskovsky V., Foster K.R. (2025). Metabolic ecology of microbiomes: Nutrient competition, host benefits, and community engineering. Cell Host Microbe.

[165] Merenstein D., Pot B., Leyer G., Ouwehand A.C., Preidis G.A., Elkins C.A., Hill C., Lewis Z.T., Shane A.L., Zmora N. (2023). Emerging Issues in Probiotic Safety: 2023 Perspectives. Gut Microbes.

[166] Varma S., Gulati K.A., Sriramakrishnan J., Ganla R.K., Raval R. (2025). Environment signal dependent biocontainment systems for engineered organisms: Leveraging triggered responses and combinatorial systems. Synth. Syst. Biotechnol..

[167] Bianconi I., Aschbacher R., Pagani E. (2023). Current uses and future perspectives of genomic technologies in clinical microbiology. Antibiotics.

[168] Mruk-Mazurkiewicz H., Kulaszyńska M., Jakubczyk K., Janda-Milczarek K., Czarnecka W., Rębacz-Maron E., Zacha S., Sieńko J., Zeair S., Dalewski B. (2023). Clinical relevance of gut microbiota alterations under the influence of selected drugs—Updated review. Biomedicines.

[169] Gilbert J.A., Azad M.B., Bäckhed F., Blaser M.J., Byndloss M., Chiu C.Y., Chu H., Dugas L.R., Elinav E., Gibbons S.M. (2025). Clinical translation of microbiome research. Nat. Med..

[170] Diacova T., Cifelli C.J., Davis C.D., Holscher H.D., Kable M.E., Lampe J.W., Latulippe M.E., Swanson K.S., Karl J.P. (2025). Best practices and considerations for conducting research on diet–gut microbiome interactions and their impact on health in adult populations: An umbrella review. Adv. Nutr..

[171] Sahoo D., Rodriguez E., Nguyen K., Chintapula U. (2025). Probiotic bacteria as therapeutics and biohybrid drug carriers: Advances, design Strategies, and future outlook. ACS Appl. Bio Mater..

[172] Mousavinasab F., Karimi R., Taheri S., Ahmadvand F., Sanaaee S., Najafi S., Halvaii M.S., Haghgoo A., Zamany M., Majidpoor J. (2023). Microbiome modulation in inflammatory diseases: Progress to microbiome genetic engineering. Cancer Cell Int..

[173] Dey S., Sankaran S. (2024). Engineered Bacterial Therapeutics with Material Solutions. Trends Biotechnol..

[174] Radovanovic M., Kekic D., Gajic I., Kabic J., Jovicevic M., Kekic N., Opavski N., Ranin L. (2023). Potential Influence of Antimicrobial Resistance Gene Content in Probiotic Bacteria on the Gut Resistome Ecosystems. Front. Nutr..

[175] Spacova I., Binda S., Ter Haar J.A., Henoud S., Legrain-Raspaud S., Dekker J., Espadaler-Mazo J., Langella P., Martín R., Pane M. (2023). Comparing technology and regulatory landscape of probiotics as food, dietary supplements and live biotherapeutics. Front. Microbiol..

